#  Literature-informed gene extraction and ranking for multimodal data fusion

**DOI:** 10.1093/bib/bbag348

**Published:** 2026-07-05

**Authors:** Marietta Hamberger, Silke D Werle, Johann M Kraus, Hans A Kestler

**Affiliations:** Institute of Medical Systems Biology, Ulm University, Albert-Einstein-Allee 11, 89081 Ulm, Germany; Institute of Medical Systems Biology, Ulm University, Albert-Einstein-Allee 11, 89081 Ulm, Germany; Institute of Medical Systems Biology, Ulm University, Albert-Einstein-Allee 11, 89081 Ulm, Germany; Institute of Medical Systems Biology, Ulm University, Albert-Einstein-Allee 11, 89081 Ulm, Germany; Leibniz Institute on Aging – Fritz Lipmann Institute, Beutenbergstraße 11, 07745 Jena, Germany

**Keywords:** knowledge fusion, text mining, symbolic representation, contextual ranking

## Abstract

Published biomedical experiments provide an increasingly rich collection of results and identify genes potentially involved in diverse biological mechanisms. However, individual studies are often confined to narrow experimental contexts and are restricted to single omics layers. Cross-study knowledge aggregation can broaden this perspective and enable the construction of global, context-aware gene rankings. Recent developments in natural language processing have made large-scale literature mining increasingly feasible. This enables the systematic extraction and fusion of symbolic knowledge from published experiments. We present pathXcite, a software that extracts genes associated with specific contexts, such as diseases or biological mechanisms from the literature, and ranks them by contextual relevance. These relevance-based gene rankings can compress a scientific context into a symbolic representation. This representation enables diverse downstream analyses, including cross-context comparisons, network-based analysis, enrichment analysis, and integration with experimental omics data. In multiple use cases, we show how our extraction and fusion strategy can be applied to uncover hidden aspects in biological data.

## Introduction

The biomedical literature is a vast and continuously growing repository of mechanistic knowledge. It describes relationships among genes, pathways, diseases, and therapeutic interventions on multiple omics levels. Much of this information is embedded in specific experimental contexts and formulated in a narrative form. As a result, it is difficult to access and systematically reuse. Researchers investigating a biological topic, such as a disease or molecular process, frequently choose manual strategies to construct gene lists from heterogeneous sources [1–3]. However, this process is time-consuming, difficult to reproduce, and has limited scalability [4]. Furthermore, it typically does not provide a graded assessment of gene relevance and only implicitly integrates evidence across different studies. Advances in natural language processing (NLP) have made it increasingly feasible to systematically mine this unstructured data. Methods such as named entity recognition (NER) and relation extraction allow the transformation of text into structured biological representations [5–7]. Especially deep learning-based approaches have significantly improved gene recognition [8]. By incorporating contextual cues, they often outperform dictionary-based methods and can robustly handle domain-specific terminology [9–11].

Despite these advances, the extracted gene lists remain closely tied to individual publications. Each study typically discusses only a small number of genes under specific experimental conditions. The information is, therefore, fragmented and difficult to interpret at the level of a broader biological topic. To address this, gene aggregation and ranking strategies are required. Such approaches can transform evidence across studies and reveal global patterns that are not apparent from single publications. Importantly, they enable the prioritisation of genes for downstream analyses, such as multimodal data fusion, pathway enrichment, network modelling, or hypothesis generation.

Several approaches have been proposed to extract and prioritise genes from the literature. These approaches generally differ in three main aspects. First, they vary regarding the used text sources, ranging from keyword annotations (e.g. MeSH Terms) and abstracts to full-text articles [12–15]. Second, they use different information retrieval methods, including simple co-occurrence statistics or context-based approaches that consider semantic relations between biomedical entities [16, 17]. Lastly, they differ in the way that extracted genes are evaluated and ranked. Many existing methods rely on frequency-based measures that capture how often a gene is mentioned in connection with a topic [12, 14, 15]. Such approaches are simple and scalable. However, they do not distinguish between genes that are merely present and those that are specifically associated with the topic. As a result, they are prone to bias towards heavily studied genes and may overlook more specific but less frequently mentioned candidates.

A high percentage of existing work integrates literature-based information with external data sources, such as gene expression data or curated databases [18]. While incorporating additional biological knowledge can increase the robustness of gene prioritisation, it can also introduce potential biases or additional assumptions. Moreover, it reduces the traceability of the resulting scores, as relevance rankings become influenced by data sources beyond the literature itself. In contrast, purely literature-based approaches derive associations directly from published knowledge. This enables a transparent and reproducible mapping from text to gene-topic associations. Such transparency is particularly important when the resulting rankings are used to guide downstream analyses and biological interpretation. The challenge here is, therefore, not only to capture the frequency of single genes but also their relevance within the context of a particular topic.

In information retrieval, this problem is addressed by weighting schemes that combine a term’s local frequency with its global distribution across a corpus [19–21]. Such methods are commonly used for general keyword identification, as they can identify terms that are both frequent and discriminative. By analogy, in a biomedical context, gene-topic associations should be evaluated not only by their frequency but also by their specificity with respect to a broader background corpus. Building on this idea, we present pathXcite, a method that derives literature-based gene rankings using a weighting scheme that integrates local and global evidence. Our approach enables scalable, transparent, and reproducible prioritisation of genes within a defined topic corpus without relying on external sources or single omics layers. The resulting gene rankings are interpretable and directly usable in downstream analyses, including, but not limited to, multimodal data fusion, enrichment analysis, candidate gene prioritisation for feature selection, network analysis, and further exploratory studies. In the following sections, we describe the individual components of the framework and the associated configuration options, validate the performance of pathXcite, and demonstrate its application in several use cases.

## PathXcite

PathXcite is a stand-alone graphical user interface (GUI) application that integrates literature curation, gene extraction and ranking, and downstream analysis into a single modular pipeline. The tool supports both manual and automated processes, offering flexibility for exploratory analysis while ensuring efficient processing through automated steps. In the following sections, we describe the main components of the workflow.

### Literature collection

At the beginning of the pipeline, the researcher formulates a topic or a question for which literature, and subsequently genes, are retrieved. PathXcite provides an embedded web browser that allows users to navigate PubMed and PubMed Central (PMC) or browse the web freely within a familiar interface (Fig. 1.1a). We recommend restricting the search using logical connectives (AND and OR) or specifying whether the search term should appear in the title. Unlike fully automated retrieval methods, this user-driven approach ensures that researchers retain full control over literature selection. Found articles can be selected manually or added in batches by extracting all PubMed and PMC identifiers from the currently loaded webpage. This extraction occurs locally within the browser without automated web crawling or indexing beyond the user’s actions. For each article, the available text, as well as bibliographic metadata, are retrieved through the E-utilities provided by NCBI (National Center for Biotechnology Information) [22]. If the article is indexed in PubTator^3^, its annotated genes are retrieved using the database’s API service [23, 24]. All data collected are stored locally in an SQLite3 database, ensuring persistence, transparency, and reproducibility (Fig. 1.1b). Rather than relying on the exhaustive inclusion of topic-related literature, users retain full control over both the selection and the number of articles used in downstream analyses (Fig. 1.2c). After collecting potentially relevant articles, pathXcite offers visualisation options that enable users to manually filter the articles for the most relevant ones. One corpus-wide option is to obtain a deeper understanding of a topic’s popularity across time. On a more granular level, users can view the prevalence of genes across an article’s sections. This is especially helpful when individual articles predominantly mention genes in the references or introduction instead of their main findings (Fig. 2).

**Figure 1:**
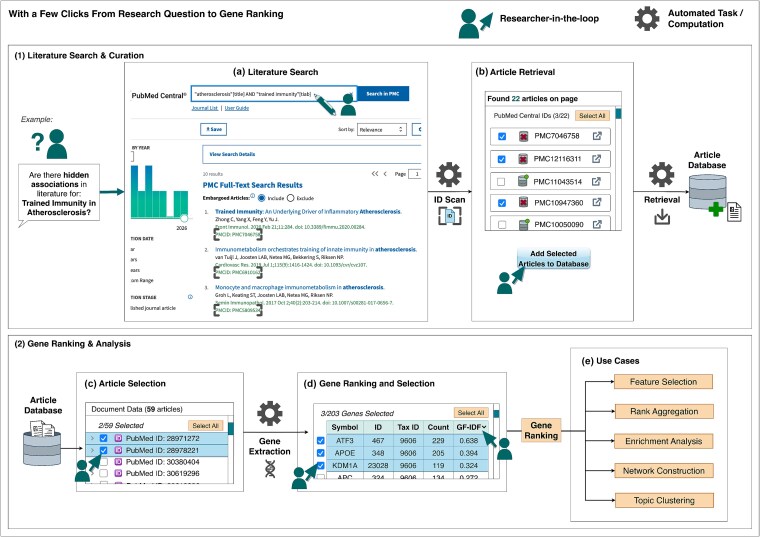
The graphical user interface of pathXcite guides users from literature curation through gene extraction and subsequent analysis, using a structured, researcher-in-the-loop workflow.

**Figure 2:**
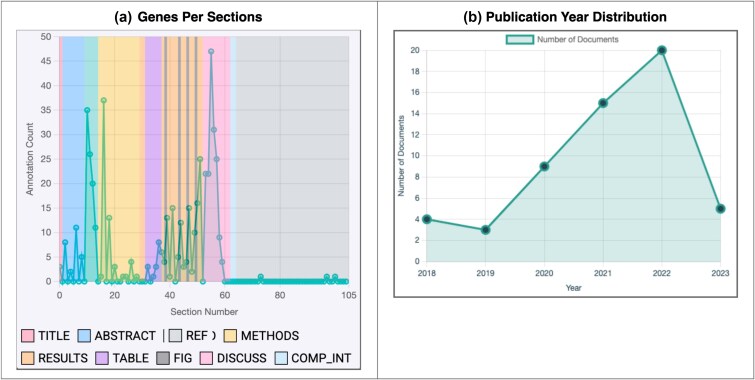
Interactive plots created in pathXcite. The tool provides a document insights module, which, for example, shows (a) the distribution of found genes over an article’s sections or (b) the distribution of selected articles per publication year. Other options not shown here include bar plots to show the most frequent journals or MeSH terms in the articles. All plots are interactive and can be exported to an HTML file.

### Gene ranking and selection

After article selection and gene extraction, pathXcite compiles an initial candidate gene list (Fig. 1.2d). The user can then create a gene ranking for subsequent analysis. In pathXcite, each gene is listed in a table with its standardised gene ID alongside the annotation frequency, representing the number of times the gene is mentioned in the selected literature, and the exact textual context. If applicable to the user’s research question, the list can be filtered to include only those gene entries that are associated with a custom set of species.

Many existing ranking methods rely on frequency-based methods [19]. However, annotation frequency alone can overemphasise genes that are widely studied and frequently mentioned in the literature, even if they are not particularly relevant to the specific topic under investigation. We, instead, define a weighting scheme that accounts for both local and global evidence, which we call Gene Frequency-Inverse Document Frequency (GF-IDF). Here, we adjust the frequency of a gene within the selected corpus relative to its prevalence in the entire PubTator^3^ database. Thus, it emphasises genes that are contextually relevant within a selected corpus, rather than those frequently mentioned in the broader literature [19–21].

We define the GF-IDF of a gene $g$ as follows:


(1) \begin{eqnarray*}& \mathrm{GF-IDF}(g; A, P) = \mathrm{GF}_{A}(g) \cdot \mathrm{IDF}_{P}(g)\end{eqnarray*}

with:


(2) \begin{eqnarray*} \mathrm{GF}_{A}(g) &= \frac{\sum_{a \in A} m(g, a)}{\sum_{a \in A} \sum_{g^{\prime} \in \Gamma(A)} m(g^{\prime}, a)} \end{eqnarray*}

(3) \begin{eqnarray*} \mathrm{IDF}_{P}(g) &= \ln \left( \frac{|P| + 1}{\mathrm{df}_{P}(g) + 1} \right) \end{eqnarray*}

so that:


(4) \begin{eqnarray*}& \mathrm{GF}-\mathrm{IDF}(g; A, P) = \frac{\sum_{a \in A} m(g, a)}{\sum_{a \in A} \sum_{g^{\prime} \in \Gamma(A)} m(g^{\prime}, a)} \cdot \ln \left( \frac{|P| + 1}{\mathrm{df}_{P}(g) + 1} \right)\end{eqnarray*}

where $A$ represents set of selected articles; $P$: set of all annotated articles available in the PubTator^3^ database; $m(g, a)$: number of mentions of gene $g$ in article $a$; $\Gamma (A) = \{\, g^{\prime} \mid \sum _{a \in A} m(g^{\prime}, a)> 0 \,\}$: set of genes mentioned at least once in $A$, with $g^{\prime}$ referring to a single gene in this set; and $\mathrm{df}_{P}(g) = \bigl | \{\, p \in P \mid m(g, p)> 0 \,\} \bigr |$: number of articles in $P$ that mention gene $g$ at least once.

Depending on the type of subsequent analyses, the user can decide whether to rank the genes found based on annotation frequency or GF-IDF scores. Moreover, the user can specify the number of top-ranking genes to include in the final ranking. The resulting gene ranking can be exported with the corresponding scores (Fig. 1.2d).

### Enrichment analysis

While there exist various ways to further analyse the constructed gene rankings (Fig. 1.2e; see also Section Use cases), our current version of pathXcite already provides an interactive over-representation analysis (ORA) module. To perform these analyses, users can select from over $200$ available gene set libraries, depending on the need for their specific research question. These libraries map gene sets to a wide range of concept types. Options include, but are not limited to, pathways (e.g. KEGG [25], Reactome [26]), molecular processes (e.g. Gene Ontology (GO) [27]), phenotypes (e.g. Human Phenotype Ontology [28]), or drug signatures (e.g. DSigDB [29]).

Within pathXcite, ORA is performed using Fisher’s exact test, with the background gene universe defined as all genes occurring in the PubTator^3^ database. No hard-coded significance thresholds are applied. The user can select their preferred multiple-testing correction method and sort the results interactively. After selecting the appropriate gene set library, over-represented terms are identified and presented in an interactive table. For each term, a commonly used statistical measures are provided, such as the number of overlapping genes, adjusted $P$-value, odds ratio, and combined enrichment score. These measures aid in the interpretation and prioritisation of relevant biological signals. The ranking of terms can be based on any of these measures, depending on the analytical objective. To identify the most significantly enriched terms, we recommend using the adjusted $P$-value. When the magnitude of enrichment is of greater interest, the overlap proportion ($p_{\mathrm{overlap}}$) and odds ratio ($\rm OR$) may be more informative, which are defined as


\begin{align*} & p_{\mathrm{overlap}} = \frac{n_{\mathrm{shared}}}{n_{\mathrm{term}}}, \qquad \mathrm{OR} = \frac{n_{\mathrm{shared}}\cdot n_{\mathrm{neither}}}{n_{\text{only term}}\cdot n_{\text{only list}}}, \end{align*}

where $n_{\mathrm{shared}}$: number of genes shared by the term, the gene list and the background genes; $n_{\mathrm{term}}$: total number of genes in the term; $n_{\mathrm{onlyTerm}}$: number of genes in the term but not in the list; $n_{\mathrm{onlyList}}$: number of genes in the list but not in the term; $n_{\mathrm{neither}}$: number of genes in neither set.

### Technical implementation

PathXcite is implemented as a stand-alone, cross-platform desktop application (Python 3.12), using PyQt5 for the GUI and embedded HTML/JavaScript components for interactive visualisations. All data retrieved are stored locally in a SQLite3 database to ensure reproducibility, session persistence, and offline access.

The tool is designed to operate within the ethical access limits of the external services it employs. The selection of literature is user-driven, and no automated crawling or indexing is performed beyond what a user manually initiates. All external queries, like those to NCBI E-utilities and PubTator^3^, respect their respective usage policies and rate limits [23, 24].

Compatible with Windows, macOS, and Linux, no pre-existing Python environment is necessary: all dependencies and the appropriate Python interpreter are bundled and installed automatically via an installation script. This encapsulated set-up ensures ease of use for researchers of any level of technical knowledge and consistency across systems. The full source code of pathXcite, installation instructions, and documentation are available on GitHub and Zenodo, further details about computation time can be found in the supplementary material (Section S1.1).

## Comparison of gene extraction and normalisation strategies

NER and named entity normalisation (NEN) form the technical foundation of literature-derived gene rank construction [5–7]. NER detects gene mentions in text, while NEN links these mentions to standardised unique identifiers, such as NCBI Gene IDs. Because pathXcite transforms dispersed textual evidence into structured gene sets, the quality of both steps directly affects the outcome of downstream analyses.

**Figure 3:**
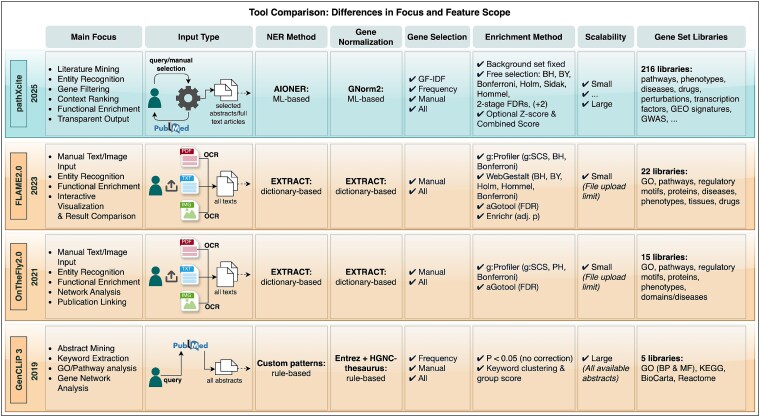
Comparison of four approaches for literature-based gene list generation and downstream enrichment analysis: tools are evaluated based on main use cases, input type, NER method, gene normalisation and selection strategies, enrichment methods, scalability, and access to gene set libraries. Abbreviations: ML, Machine-Learning; NER, Named Entity Recognition; GF-IDF, Gene Frequency-Inverse Document Frequency; BH, Benjamini-Hochberg; BY, Benjamini-Yekutieli; FDR, False Discovery Rate; GO BP, Gene Ontology Biological Process; GO MF, Gene Ontology Molecular Function; OCR, Optical Character Recognition.

To perform these tasks, pathXcite uses PubTator^3^ [24], which combines the machine learning-based AIONER system [30] for NER with GNorm2 [31] for NEN. Existing tools that also similarly support gene aggregation followed by downstream analyses are, for example, OnTheFly2.0 [12] and FLAME2.0 [14]. Both rely on a dictionary-based system, namely the EXTRACT tagging service [32]. GenCLIP3 [15] represents another tool with a similar purpose, but it uses a rule-based approach for NER and NEN. Although all listed systems support gene-focused text mining with optional enrichment analysis, they differ substantially in annotation strategies, coverage, gene prioritisation, and downstream analysis objectives. Figure 3 summarises these differences.

Previous studies have shown that machine learning-based systems generally outperform dictionary- and rule-based approaches in the NER and NEN tasks [33]. To substantiate this, we directly compare the performance of EXTRACT [32] and PubTator^3^ [24].

For this comparison, we used the BioRED test set, a gold standard corpus containing $1{}180$ manually annotated and normalised gene mentions from $100$ PubMed articles [34]. Strict scoring criteria were applied during NER evaluation: partial matches (e.g. identifying “Interleukin 6” when the true mention is “Interleukin 6 receptor”) were counted as both false positives and false negatives in order to penalise over-specific and under-specific recognition symmetrically. NEN accuracy was calculated as the proportion of correctly normalised genes among the true positives of the NER step.

**Figure 4:**
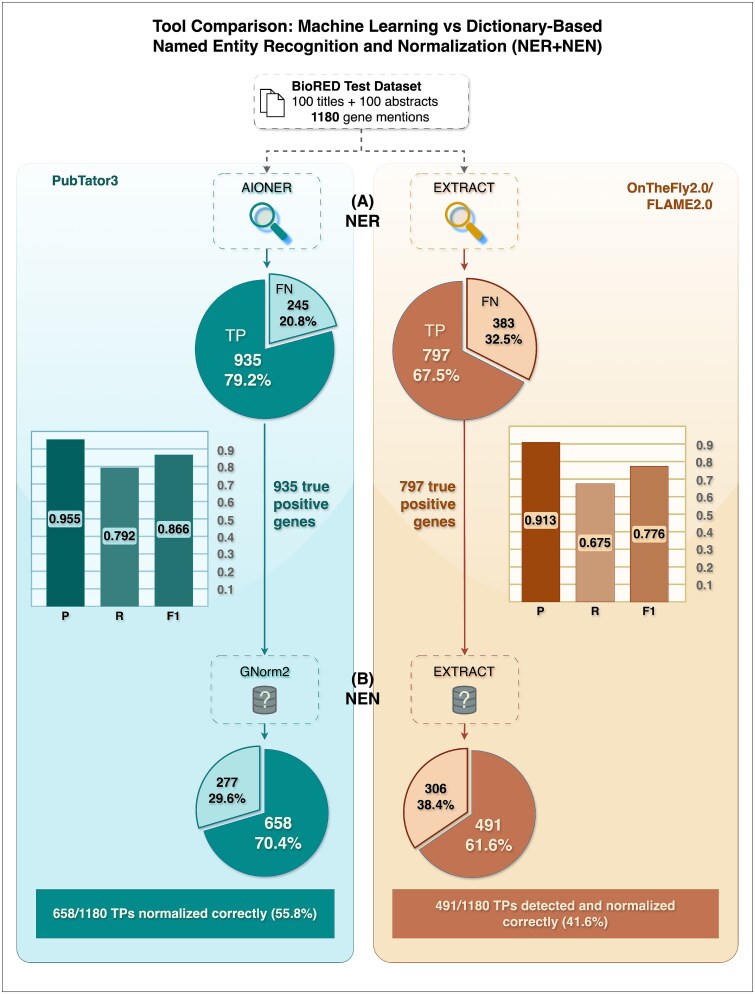
Comparison of machine learning-based (AIONER+GNorm2) and dictionary-based (EXTRACT) approaches for gene NER and NEN. Bar plots show precision (P), recall (R), and F1-score for NER (1) and NEN (2) tasks across both systems. Pie charts display the proportions of true positives (TP) and false negatives (FN), averaged over five repetitions, for both NER and NEN stages out of $1{}180$ gene mentions in the test dataset. AIONER+GNorm2 consistently outperforms EXTRACT across all evaluation metrics, achieving higher recall and F1-scores in both tasks, with a notably lower false negative rate.

In the NER evaluation, AIONER achieved higher recall than EXTRACT ($79.2\%$ versus $67.5\%$) while maintaining comparable precision, resulting in $138$ additional true positive recognitions (Fig. 4a). NEN accuracy was similar across methods, with a slight advantage for GNorm2 when applied to AIONER-derived mentions (Fig. 4b). Overall, the machine learning-based pipeline improved end-to-end coverage primarily through increased recall in the NER step. As pathXcite derives gene rankings from aggregated literature evidence, incomplete recognition directly propagates to the ranking stage. This can potentially bias results towards frequently mentioned genes while omitting less prominent but relevant candidates. The improved recall, therefore, enhances both the completeness and robustness of the resulting gene rankings. Based on these findings, we adopted PubTator^3^ within pathXcite.

## Performance evaluation

Gene set construction. Literature-based gene list generation transforms unstructured textual evidence into structured gene collections associated with a defined topic. The quality and interpretability of these collections depend on two key factors: the definition of the literature corpus and the strategy used to prioritise genes within it. Increasing the number of articles can improve coverage of topic-relevant signals but may also introduce noise through the inclusion of broadly discussed or only weakly related genes. Consequently, a proper gene ranking strategy should remain robust to variations in corpus size.

Many gene set libraries cite manually curated literature as references. In KEGG, we found that pathway entries are supported by an average of $11$ referenced articles, making this database suitable for benchmarking.

**Figure 5:**
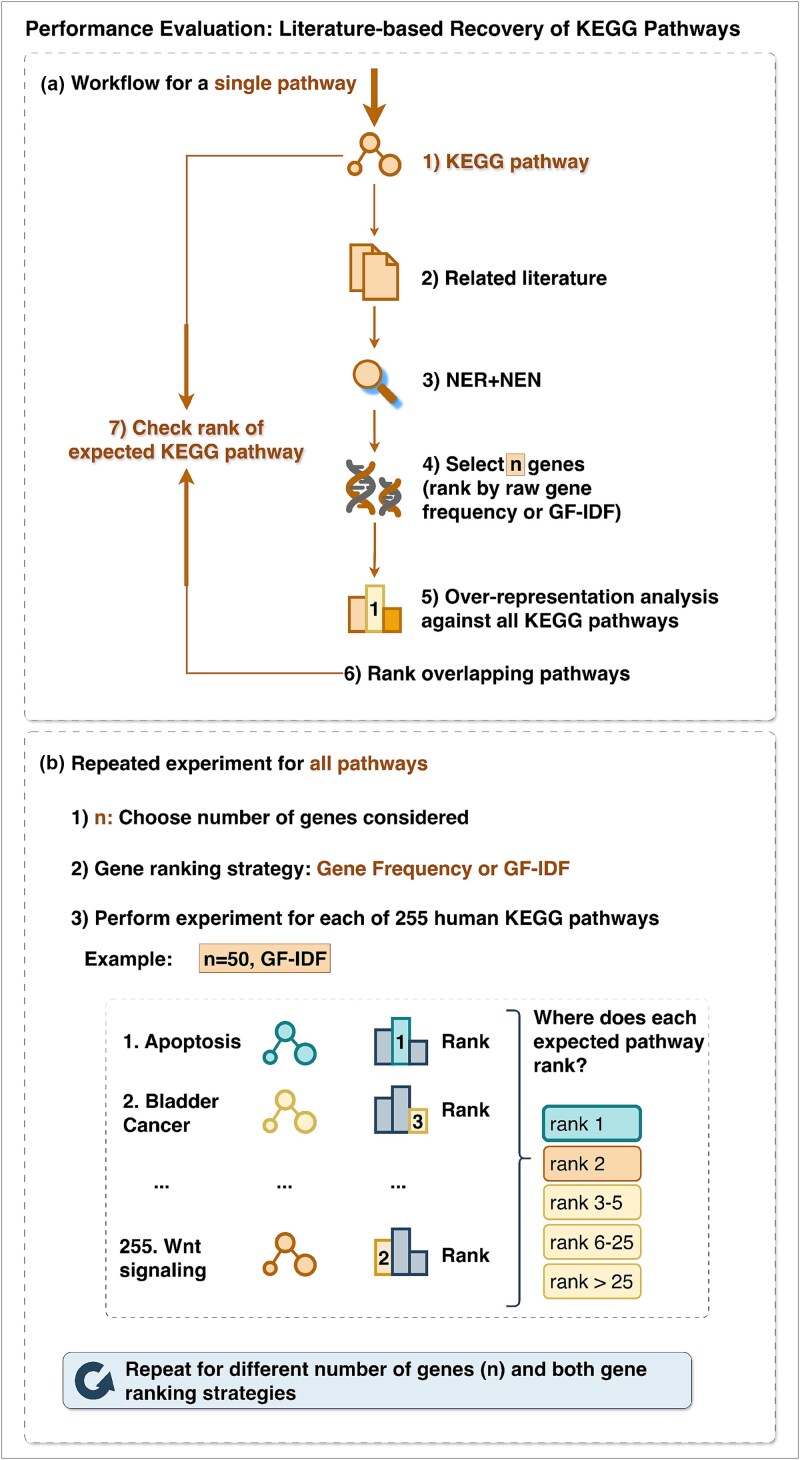
Performance evaluation. Workflow and evaluation strategy for literature-based recovery of KEGG pathways. (a) For each KEGG pathway, we retrieved referenced literature, extracted gene mentions (NER and NEN), selected genes (by one of four tested strategies), and performed ORA to assess whether the original pathway could be recovered. (b) This process was repeated for $255$ human KEGG pathways using varying gene list sizes and both gene ranking strategies. Recovery performance was evaluated based on the rank and significance of the expected KEGG pathway among enrichment results.

To prove the performance of pathXcite, we evaluated whether gene lists derived from KEGG reference articles are sufficient to recover the associated pathway in enrichment analysis (Fig. 5a). More specifically, we ask: if we apply our method only to the literature cited by a pathway, can we reconstruct that pathway with the resulting genes through ORA? This evaluation is based on the assumption that the literature curated for each KEGG pathway reflects its underlying gene composition. Consequently, gene rankings derived from these references should recover the corresponding pathway genes if relevant signals are correctly captured and prioritised. We, therefore, assess how well literature-derived gene sets overlap with the curated pathway definitions. We use ORA to quantify this overlap in a statistically defined manner. In this setting, enrichment significance directly reflects the agreement between the selected genes and the pathway gene set. At the same time, this set-up demonstrates how rank-derived gene lists can be used in downstream analyses such as enrichment testing. Additionally, this setting provides a controlled test using small, high-quality article sets and enables us to quantify how gene selection, ranking strategies and gene list size influence pathway recovery. Of the $320$ human pathways included in the 2022 KEGG database, $255$ are associated with reference literature and contain at least one human gene mention. For each pathway, we retrieved its curated references and extracted human gene mentions from the PubTator^3^ database. After filtering out pathways that did not mention human genes or were not yet supported by literature references, we retained $255$ pathways, each linked to an average of $60$ unique human genes.

Because downstream analyses depend heavily on the input gene list, we compared four gene selection strategies:

- All extracted genes: use the full set of (human) genes mentioned in the article set.
- Random sampling: select random gene subsets of varying sizes to assess robustness.
- Raw frequency-based selection: prioritise genes based on raw annotation frequency within the set of articles.
- GF-IDF-based selection: apply our previously introduced measure to evaluate whether context-aware filtering improves enrichment quality.

For each input gene list, we then performed ORA (Fisher’s exact test; correction: Benjamini–Hochberg). This returned all pathways sharing at least one gene with the input set, along with statistical significance scores. We ranked the resulting pathways by their adjusted $P$-values to evaluate how well the original (i.e. expected) KEGG pathway was recovered. To assess the influence of gene selection strategy on pathway recovery, we computed three complementary measures:

- Rank of the expected pathway: the position of the target KEGG pathway in the ranked results.
- Statistical significance: whether the expected pathway was significantly enriched (adjusted $P$-value $<.05$).
- MRR: a statistical measure of overall ranking performance, calculated as the average of the reciprocal ranks ($\frac{1}{\mathrm{rank}}$) of the expected KEGG pathways across all test cases. Higher MRR values indicate better recovery performance.

To facilitate comparison between strategies, we binned the results into rank categories.

- **
$\mathrm{Rank} = 1$ :** optimal performance (the expected pathway is the most enriched).
- **
$\mathrm{Ranks} =2$ , $3-5$, $6-25$, or $>25$:** additional accuracy thresholds to measure robustness.

**Figure 6:**
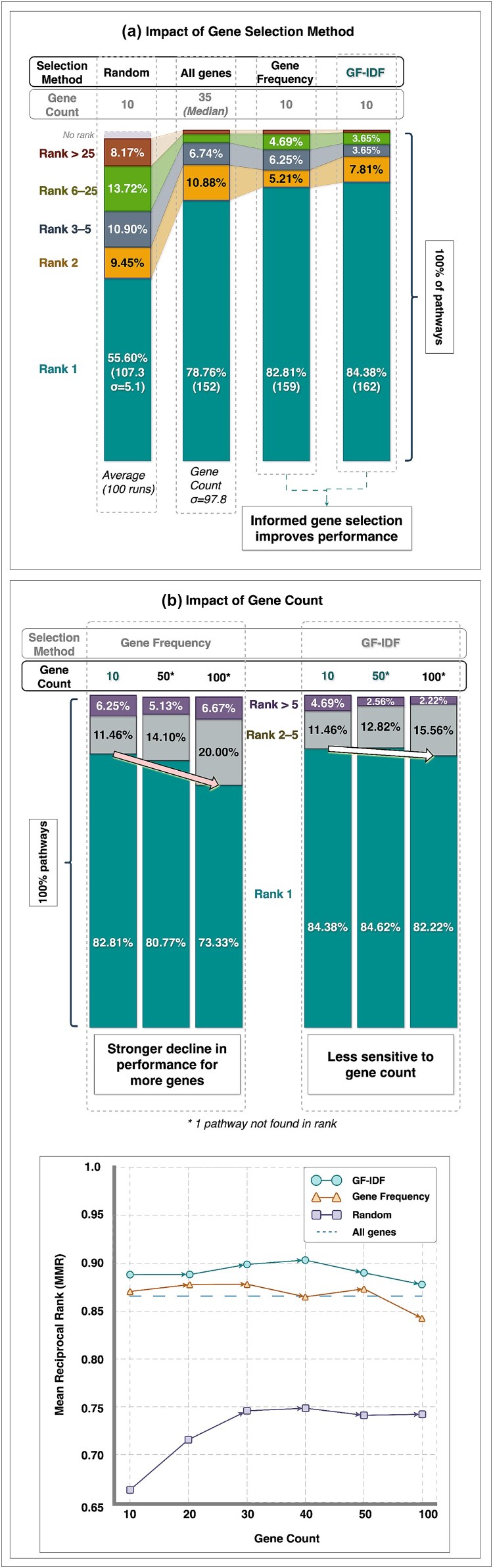
Performance evaluation. Impact of gene selection strategy and gene list size on pathway recovery accuracy. (a) Comparison of gene selection methods (Random, All genes, Frequency, andGF-IDF) using a fixed gene count ($n = 10$). GF-IDF achieves the highest proportion of $\mathrm{Rank} = 1$ recoveries. (b) Effect of increasing gene list size ($n \in \{10, 50, 100\}$) for frequency-based and GF-IDF methods. GF-IDF maintains higher and more stable performance across sizes, while frequency-based selection declines more strongly with larger lists.

This allowed us to assess the impact of the gene selection strategy under varying conditions on both the position and the statistical significance of expected pathways. We observed that the strategy used to select and rank genes strongly influences pathway recovery performance. When controlling for gene list size ($n=10$), GF-IDF consistently outperformed other methods, recovering the expected pathway at $\mathrm{Rank} = 1$ in $84.4\%$ of cases (Fig. 6a). Frequency-based selection achieved comparable but slightly lower performance in $82.8\%$, while random subsets performed considerably worse, with only $55.6\%$ of the pathways recovered in the top rank. Using all available genes from the literature performed moderately at $78.8\%$, suggesting that while comprehensive extraction captures signal, it also includes distracting noise. These differences are further supported by MRR values. GF-IDF yielded the highest MRR ($0.888$), closely followed by frequency-based ranking ($0.869$), with the full gene list following slightly behind ($0.865$). Random selection again showed the weakest performance ($0.663$).

Since the number of unique genes mentioned in a pathway’s literature ranged from fewer than 10 to over $400$, we also examined how gene list size impacts pathway recovery (Fig. 5b). Therefore, we performed random sampling of $n \in \{10, 20, 30, 40, 60, 80, 100\}$ genes from the entire list. This was repeated $100$ times for each selection to account for variability. In addition, for frequency-based methods (namely ranking by raw annotation frequency and GF-IDF), we also chose the top $n \in \{10, 20, 30, 40, 60, 80, 100\}$ genes, but ranked by their respective scores.

Here, GF-IDF showed a high degree of stability across all tested sizes, with $\mathrm{Rank} = 1$ recovery consistently above $81.9\%$ and an MRR that peaked at $0.903$ for $n=40$ genes (Fig. 6b). Even as the gene list grew to $n=100$, performance remained strong, with minimal decline in ranking accuracy. In contrast, frequency-based ranking was more sensitive to increasing gene list size. While its performance was initially high at $n=10$ ($82.8\%$  $\mathrm{Rank} = 1$, $\mathrm{MRR} = 0.869$), it declined steadily as additional genes were included, reaching $73.3\%$  $\mathrm{Rank} = 1$ and an MRR of $0.842$ at $n=100$. Random selection was only marginally affected by gene list size. Although MRR improved from $0.663$ at $n=10$ to $0.742$ at $n=100$, the overall performance remained substantially lower than the GF-IDF and the raw frequency-based approach.

These results suggest that the accuracy or the quality of other downstream analyses depends not only on the presence of relevant genes but also on how effectively they are ranked before selection. Methods like GF-IDF that incorporate contextual relevance appear to be better suited to highlight meaningful biological signals within literature-derived gene lists. Furthermore, GF-IDF is able to accommodate larger gene sets without a strong performance deterioration. The drop in MRR indicates that as gene lists expand, frequency alone is insufficient to filter out less relevant entries, reducing the method’s precision in comparison to a more context-based selection. The slight improvement at higher $n$ for random sampling of genes can be explained by the inclusion of more relevant, yet also noisy, genes. This reinforces the importance of using informed ranking, especially when working with large and potentially noisy gene sets.

Our proof of concept demonstrates that literature-derived gene rankings can effectively be used to recover the genes in KEGG pathways. The performance improves when paired with informed gene selection strategies like GF-IDF weighting.

Scalability. Next, we evaluated the scalability of our approach in a more realistic setting. Therefore, we applied pathXcite to a broad range of diseases using semi-automatically retrieved literature. This set-up reflects how researchers might generate topic-specific gene collections from large corpora.

The KEGG database currently contains $2828$ diseases and $1210$ of these are mapped to at least one pathway. For each mapped disease, we retrieved up to $1000$ PubMed articles with the disease name in the title and kept only those that mentioned human genes. Eight diseases did not have such articles.

Based on our proof-of-concept finding that the top 10 GF-IDF genes already provide reliable enrichment, we kept only diseases with at least 10 extractable genes. This yielded $1142$ diseases, each linked to at least one KEGG pathway. For each, we used the 10 most relevant genes based on GF-IDF ranking for enrichment. To examine the effect of article count, we binned diseases by the number of available articles. The groups required at least $a\in \{10, 20,..., 50, 100, 200,..., 1000\}$ articles, plus an additional group that used all available articles up to $1000$ per disease.

**Figure 7:**
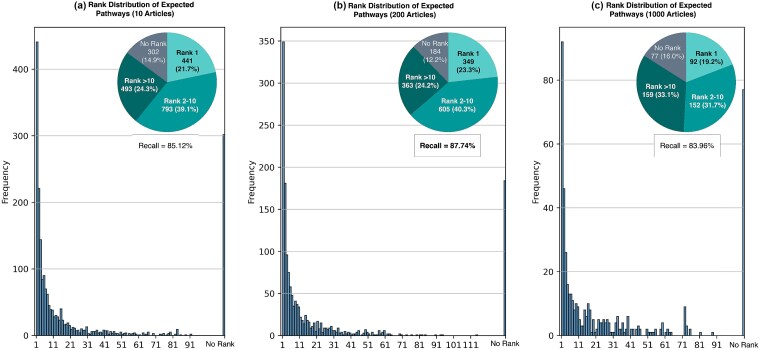
Performance evaluation. Distribution of rank positions for expected KEGG pathways across different article count thresholds. Each plot shows the distribution of the median rank of disease-associated pathways found through enrichment. Expected pathways are typically ranked within the top four results, with median ranks increasing with $1000$ articles. Ranking performance becomes more dispersed as article counts increase. Using $200$ articles showed the best balance.

First, we analysed the fraction of known KEGG pathways for each disease that were found to be enriched (recall) and its rank (Fig. 7a–c).

Thereby, we observed that even with only 10 articles, recall was high ($\approx 85.12\%$) and increased with more articles, peaking at $500$ ($\approx 88.44\%$). However, this recall gain was accompanied by shifts in ranking behaviour. The expected pathways were consistently highly ranked across thresholds, often in the top four (median rank). The most concentrated top-ranking results were observed with smaller article sets. In contrast, larger sets (e.g. $1000$ articles) achieved a wider pathway coverage but showed greater rank dispersion. Instead, median adjusted $P$-values of expected pathways improved steadily with more articles, reaching $\approx 0.002$ at $1000$ articles and $\approx 0.001$ when all retrieved articles were used, but were already significant with 10 articles (median $\approx 0.013$).

These results imply that a well-prioritised gene set can still yield meaningful enrichment. Recall can be improved by larger article sets, but this process will also introduce greater rank dispersion due to the inclusion of less relevant genes. Overall, the best balance between recovery and ranking was achieved with $200$ articles, and performance with only 10 articles remained strong, supporting the method’s usefulness for rare or understudied diseases. Larger literature sets also produced more statistically robust results. However, this comes with a trade-off. Although the adjusted $P$-value is improved, it lowers the median rank of expected pathways, reflecting increased noise in ranking despite stronger statistical support.

**Figure 8:**
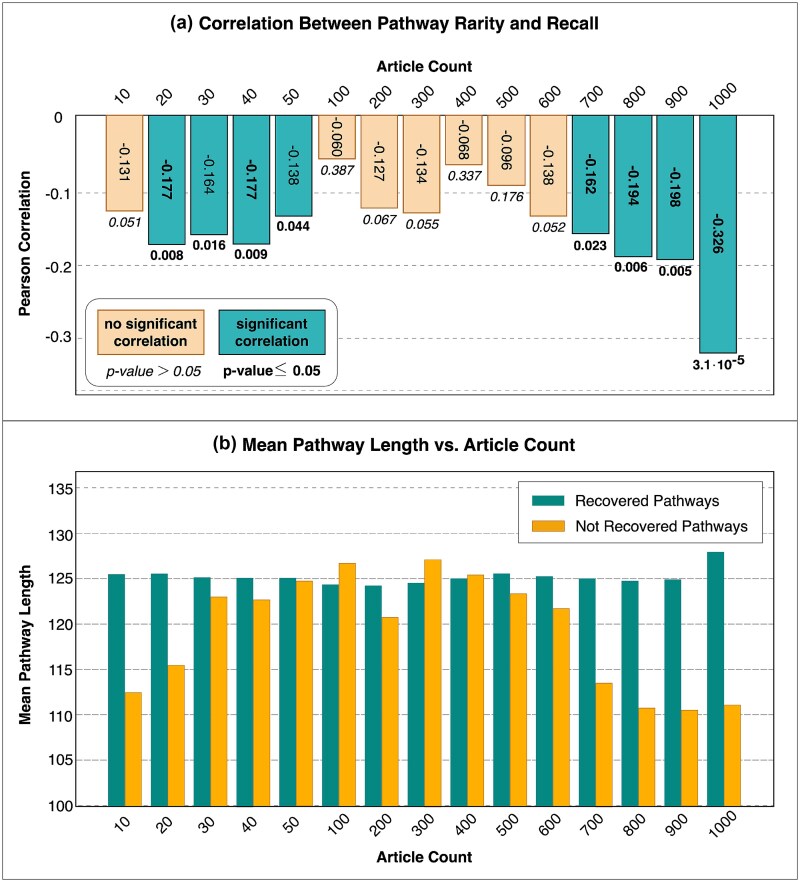
Performance evaluation. (a) Correlation between pathway recall and gene rarity across article count levels. The bar plot shows Pearson correlation coefficients and $P$-values between recall and average gene rarity of each pathway. Recall is defined as the proportion of disease-related pathways that were successfully recovered. Significant negative correlations appear at low ($\leq 50$) and high ($\geq 700$) article counts, indicating a bias towards pathways composed of more common genes. Correlations in the mid-range ($100$–$600$ articles) are not statistically significant, suggesting a more balanced representation of pathway types in that range. (b) Mean lengths of recovered versus not recovered pathways across article count thresholds. The bar plots compare mean gene counts of expected KEGG pathways that were recovered versus those missed. Low article counts: recovered pathways tend to be longer, indicating a bias towards more generic pathways. Mid-sized article sets: length difference narrows, reflecting a more balanced enrichment. High article counts: bias reappears. “Recovered” pathways are defined as sharing at least one gene with the input set and, therefore, appearing in the enrichment ranking.

At low article counts (e.g. 10 articles), recovered pathways were typically larger, with a median size of $106$ genes and a mean of $125.5$, compared with $88$ and $112.5$ for those not recovered (Fig. 8). This indicates a bias towards larger, more generic pathways when gene overlap is limited. As the count of articles increased, the size difference narrowed and briefly reversed: at $500$ articles, recovered pathways had a median/mean size of $106$/$125.6$, while those not recovered measured $114$/$123.4$. By $1000$ articles, the trend reverted, and the retrieved pathway sizes were again larger ($107$/$127.9$ versus $92$/$111.1$), suggesting a non-linear effect driven by the fixed ten-gene input.

With few articles, the gene pool is small, and key genes may be missed. With more articles, GF-IDF has richer context and can capture smaller, more specific pathways. Yet, once the pool grows too large, restricting inputs to ten genes compresses the signal and again favours more generic pathways. These biases were strongest at low ($<100$) and high ($>700$) article counts, while mid-range counts (100–600) showed no significant Pearson correlations ($P$-value $\geq .05$) between pathway recall and gene rarity and thus more balanced recovery (Fig. 8a). Finally, we evaluated how consistently each pathway was detected across disease and article count levels. Because a pathway can be associated with many diseases, we define stability as the frequency with which it appears in the disease-specific enrichment results. Many pathways were detected reliably, but others showed high variability. The least stable pathways were typically large and generic, such as signalling or immune-related pathways (Fig. 8b).

## Use cases

Gene rankings derived from the literature provide a mapping from a concept, such as a topic, disease, or biological process, to a ranked list of genes. This yields a general-purpose symbolic representation that can be directly integrated into several potential applications. In the following, we describe selected applications for pathXcite-retrieved gene rankings.

The following use cases illustrate that literature-derived gene prioritisation is not limited to a single downstream analysis, but can serve as a flexible intermediate representation for diverse knowledge-guided workflows.

### Use case 1: literature-guided feature selection

In this use case, we demonstrate how literature-derived gene lists can be used as feature priors in small-sample transcriptomic classification. We compare their performance against gene lists derived from curated databases, top differentially expressed genes (DEGs), and random gene sets.

We used an expression dataset (GSE313740) from peripheral blood mononuclear cells of subjects exposed to normoxia versus hypobaric hypoxia. The dataset includes samples collected under normoxic conditions ($t_{0}$, eight samples) and during acute hypoxia at three timepoints ($t_{1}$: $12$ h, $t_{2}$: $24$ h, $t_{3}$: $72$ h, eight samples each). Gene-level expression values correspond to normalised counts per million across all samples.

We defined three independent binary classification tasks. Each task aims to distinguish normoxic samples ($t_{0}$) from hypoxic samples at a specific time point ($t_{1}$, $t_{2}$, or $t_{3}$). Treating each task separately allowed for the assessment of how the discriminative signal evolves over time under hypoxic exposure. Given the limited sample size, we applied a leave-one-sample-out cross-validation (LOSO-CV) set-up. For each task, one sample was held out as test data while the remaining samples were used for training. This process was repeated until every sample had been used once as a test instance. All feature selection procedures were performed strictly within the respective fold-wise training data.

Classification was performed using a nearest-centroid approach [35]. For each class, the centroid was defined as the mean expression profile of the training samples belonging to that class. Prior to classification, features were standardised by a z-score normalisation step (applied within each CV fold). Test samples were assigned to the class of the closest centroid in Euclidean distance. This simple linear classifier was chosen to focus the evaluation on the quality of the selected features rather than model complexity. In total, we compared five feature selection strategies:

- literature-derived genes,
- curated pathway/ontology term genes,
- fold-specific DEGs,
- random gene subsets,
- all genes from the dataset.

Feature sets were evaluated across a range of sizes $f_{c} \in \{1, 2,..., 85\}$. For each strategy, subsets of size $f_{c}$ were constructed whenever applicable, allowing a direct comparison of the predictive performance as a function of feature set size $f_{c}$. The maximum of $85$ was chosen, since the largest overlap between a curated term and the expression data was $85$ genes (see Table 1).

**Table 1:** Use case 1: feature selection. Lower-triangular pairwise gene overlap counts (diagonal = set size)

Set	Expr	HIF1	GO	Norm	Hyp	HighAlt
Expr	**13322**	–	–	–	–	–
HIF1	**85**	109	–	–	–	–
GO	**58**	9	77	–	–	–
Norm	**85**	19	9	137	–	–
Hyp	**376**	57	27	62	538	–
HighAlt	**153**	25	16	25	78	221

Abbreviations: Expr, expression data; HIF1, HIF-1 signalling pathway; GO, Cellular response to hypoxia (GO); Norm, literature-derived normoxia gene set ($18$ articles); Hyp, literature-derived hypoxia gene set ($72$ articles); HighAlt, literature-derived high-altitude hypoxia gene set ($27$ articles). Bold counts refer to the genes used in the analysis.

Literature-based gene rankings were constructed using pathXcite from PMC queries designed to capture different oxygenation statuses (Queries: *normoxia[title] NOT hypoxia[tiab]*; *hypoxia[title] NOT normoxia[tiab]*; *“high-altitude hypoxia”[title]*). Genes were ranked according to their GF-IDF score. For classification, the top $f_{c}$ genes from each ranked list were selected. This enabled assessing how the performance changes with increasing inclusion of lower-ranked genes.

Curated gene sets were obtained from established biological knowledge bases (KEGG [25] and GO [27]). As biological processes related to hypoxia and oxidative stress, we chose the KEGG HIF-1 signalling pathway and the GO Term Cellular Response to Hypoxia (GO Biological Process GO:0071456). For each subset size $f_{c}$, $100$ random subsets were drawn and their results averaged as the representative results for this $f_{c}$ and this term.

DEG analysis was used to derive data-driven feature sets in a fold-specific manner. For each LOSO-CV iteration, the held-out sample was excluded from DEG analysis. The corresponding results from the remaining training samples were used for feature selection. For this, genes were ranked first by adjusted $P$-value (ascending) and then by absolute log fold-change (descending). For a specific feature count $f_{c}$, the top $f_{c}$ ranked genes were then selected for that fold.

As a baseline, random gene sets of size $f_{c}$ were sampled from all available genes in the dataset. For each value of $f_{c}$, $100$ independent random subsets were generated. The classification performance was averaged across repetitions.

As an upper-bound reference, all $13{\,}322$ genes in the dataset were used as features without selection. This setting represents the maximal information scenario, also including potentially noisy or irrelevant genes as features.

Across all three classification tasks ($t_{0}$ versus $t_{1}$, $t_{0}$ versus $t_{2}$, and $t_{0}$ versus $t_{3}$), predictive performance depended strongly on the number of genes included in the model. No single feature-selection strategy consistently outperformed others across the full range of feature counts ($1$–$85$). However, gene sets derived from normoxia-related literature showed the most consistently strong performance across tasks and feature ranges. Genes from the HIF-1 signalling pathway began to perform equally well at high feature counts.

Comparison between normoxic samples ($t_{0}$) and samples after $12$ h in the hypobaric chamber ($t_{1}$) showed a generally limited separability, with accuracies not exceeding $0.63$. The best performance was obtained at low to moderate feature counts using literature-derived gene sets, particularly from normoxia-related studies, which consistently achieved accuracies between $0.56$ and $0.63$. At higher feature counts, performance differences between strategies diminished, with the HIF-1 signalling pathway emerging as the most competitive approach, albeit at modest accuracy levels ($\approx 0.5$). In contrast, using all genes performed poorly ($\approx 0.38$), and random gene sets did not reach the performance of biologically informed approaches. DEG-based selection underperformed relative to literature-derived sets across most feature counts and only approached comparable performance at larger gene sets. The fact that classification performance was limited in this first task indicates that after $12$ h the impact of hypoxic conditions has not introduced massive regulatory changes.

This changed when comparing $t_{0}$ versus $t_{2}$, $24$ h. Using all genes provided a strong and stable baseline (accuracy $\approx 0.69$ across all feature counts), indicating a more globally distributed signal. Nevertheless, gene sets derived from normoxia literature repeatedly exceeded this baseline, reaching accuracies up to $0.75$ at both low (around $10$ genes) and higher feature counts ($>50$ genes). Across much of the feature range, this strategy either matched or outperformed the full-expression model. Other strategies did not achieve comparable performance: the HIF-1 pathway and high-altitude hypoxia literature gene sets showed moderate accuracy ($\approx 0.50$–$0.63$), while random subsets, DEG-based selection, and hypoxia-related literature genes consistently remained below $0.55$. Thus, while both global and targeted approaches were effective in this task, normoxia-derived gene sets provided the most consistent improvements over baseline.

The comparison between $t_{0}$ and $t_{3}$ ($72$ h) yielded the highest overall performance and the clearest dependence on feature count. Across a broad range of small-to-moderate feature counts ($<45$), normoxia-derived literature genes consistently achieved the best performance, including the overall peak accuracy of $0.875$ at only six genes, and maintained high accuracy ($>0.8$) across much of this range. At higher feature counts ($>50$), the optimal strategy shifted: the HIF-1 signalling pathway became the best-performing approach across most of the intermediate-to-high feature range, with high-altitude hypoxia literature gene sets achieving comparable performance at the largest feature counts. In contrast, hypoxia-related literature genes, GO-derived genes, the full gene set, and random subsets consistently showed lower performance (generally $\leq 0.65$). DEG-based selection dropped below all other strategies when more than five genes were used. These results indicate that, despite strong overall separability in this task, optimal performance depends heavily on both feature count and biologically informed feature selection.

**Figure 9:**
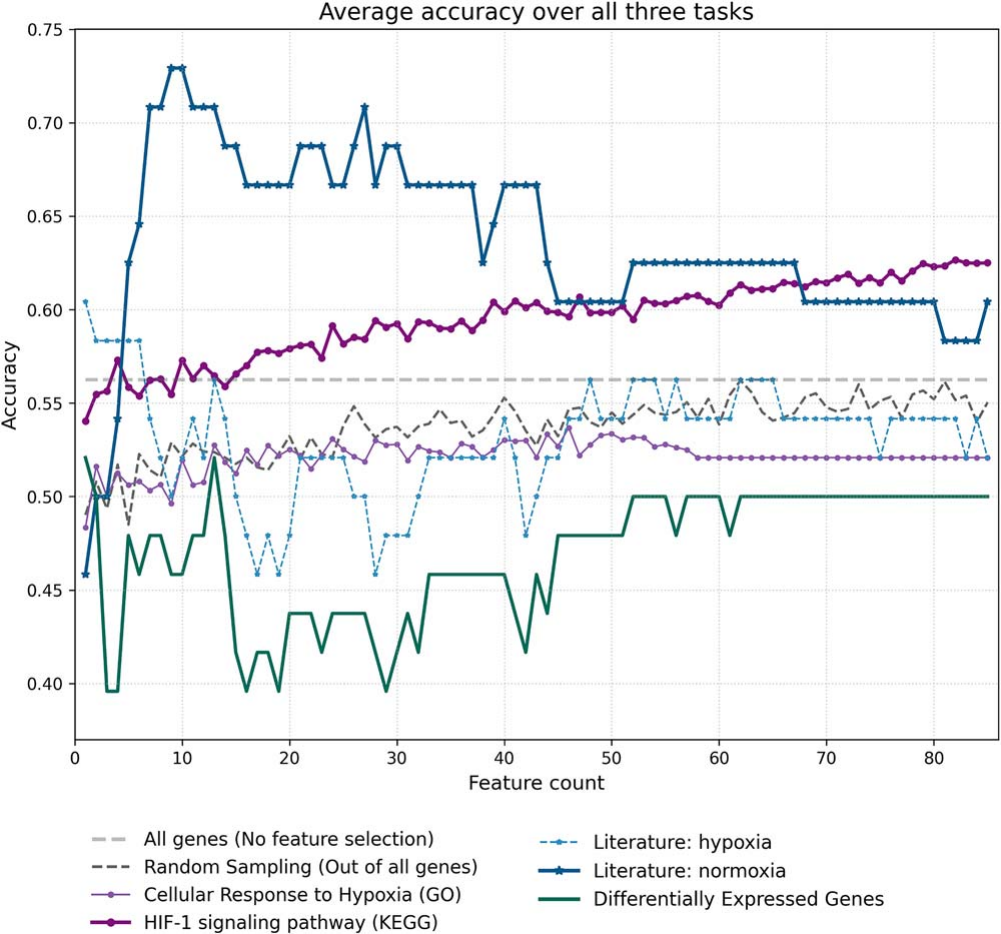
Use case 1: feature selection. Average accuracy over all three binary classification tasks using features from multiple sources including literature, pathways, and differentially expressed genes.

When performance was averaged across all tasks for each feature count, the best-performing strategy overall was the use of ranked genes derived from normoxia-related literature (Fig. 9). This approach consistently achieved the highest average accuracy across most feature counts. Only at larger feature sets ($>40$ genes) did the HIF-1 signalling pathway reach comparable performance.

In general, literature-derived and curated gene sets outperformed random selection. Random gene sets provided a stable but consistently lower baseline across all feature counts. Fold-specific DEG selection was competitive only at very low feature counts ($\leq 5$ genes), but was subsequently outperformed by other strategies as the number of features increased.

Importantly, performance did not increase monotonically with feature count. Instead, accuracy varied non-linearly, indicating that adding more genes does not necessarily improve classification performance.

### Use case 2: multi-modal rank aggregation

The construction of consensus gene rankings from multiple sources can uncover patterns that might not be visible from one input alone [36]. Integrating literature-derived evidence, based on multiple experimental sources, with differential expression data provides a multi-modal basis for gene prioritisation. For example, DEGs from own experiments can capture system-specific transcriptional changes but may include noise or lack clear biological interpretation [36]. In contrast, literature-derived signals encode more general accumulated symbolic knowledge. GF-IDF serves as a symbolic aggregation method which emphasises genes that are specifically represented within a selected corpus. However, GF-IDF alone does not account for whether these genes are active in the specific experimental context.

The multi-modal ranking aggregation enables prioritising genes that are both contextually relevant in the literature and supported by experimental evidence. This multi-modal aggregation is broadly applicable and particularly valuable when individual data sources are incomplete or noisy.

Hypermobile Ehlers-Danlos syndrome (hEDS) and Hypermobility Spectrum Disorder (HSD) are clinically related connective tissue conditions characterised by joint hypermobility, pain, and multisystem involvement. Their molecular basis remains poorly understood, and, unlike other EDS subtypes, no causative genes have been identified [37].

This lack of established genetic markers is reflected in both experimental and knowledge-based resources. Omics datasets for hEDS and HSD are limited in number and scope. Curated gene sets and database annotations remain similarly sparse. As a result, neither differential expression analysis (DEA) nor literature-based approaches alone can provide full coverage to characterise the underlying biology.

**Figure 10:**
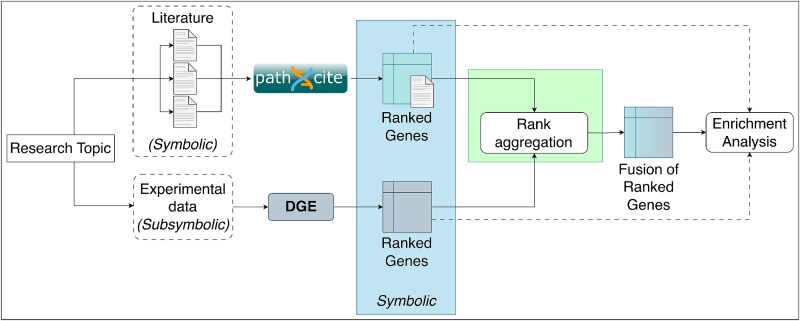
Use case 2: multi-modal rank aggregation. A research topic is explored through two parallel sources: literature, processed with pathXcite to generate GF-IDF-based gene rankings, and experimental RNA-seq data, analysed via differential gene expression (DGE). Both modalities produce ranked gene lists, which are integrated using late rank aggregation (Borda-style consensus). The resulting fused ranking is subsequently used for downstream functional interpretation via enrichment analysis.

Integrating both modalities is therefore particularly attractive in this setting. On the one hand, expression data provide direct evidence of molecular activity in a specific tissue context. On the other hand, literature-derived signals capture broader associations, including indirect links and related phenotypes. Their combination enables a more comprehensive and balanced prioritisation of candidate genes in the absence of well-defined reference sets. Figure 10 illustrates the steps of our multi-modal rank aggregation approach.

Among the available datasets, dermal fibroblast RNA-seq provides one of the few transcriptome-wide resources. The dataset used here contains RNA-seq data from $20$ hEDS patients, $20$ HSD patients, and $40$ healthy controls (GSE218012). DEA was performed on combined hEDS and HSD samples versus controls using the R packages edgeR and limma. Genes were filtered for expression, normalised using the Trimmed Mean of M-values method, and transformed to log$_{2}$ counts per million. A linear model with group as the sole factor was fitted, followed by empirical Bayes moderation. DEGs were defined as genes with absolute fold-change $\geq 1.5$ and False Discovery Rate (FDR) $\leq 0.01$, following the definition of the dataset’s original reference [38]. This yielded $1314$ DEGs from $29{\,}195$ expressed genes. DEGs were then ranked by FDR-adjusted $P$-value, followed by uncorrected $P$-value and absolute fold-change.

A literature-derived gene ranking was generated using pathXcite from PMC articles (query “‘hypermobile ehlers-danlos’[title] OR ‘hypermobile spectrum disorder’[title]”). This identified $406$ genes from all $60$ available articles, ranked by GF-IDF.

The rankings were merged using a full outer join on gene symbols. A normalised Borda-style consensus score was computed as the mean of the normalised percentile scores, with missing values set to zero [39]. This prioritises genes supported by both literature and expression data while retaining genes present in only one source. The final fused ranking was ordered by join presence, consensus score, average percentile, and average raw rank.

As an interpretive output layer, ORA was performed using the libraries GO Biological Process (2025) [27] and DisGeNet [40]. The background gene universe consisted of all genes present in either the literature-derived set or the expression dataset. GO terms with $2$–$500$ overlapping genes were retained. Statistical significance was evaluated using a hypergeometric test, $P$-values were adjusted using the Benjamini–Hochberg FDR correction. Enrichment was assessed for three gene sets:

- all $1{}314$ DEGs,
- top $50$ out of $406$ literature-derived genes,
- top $500$ out of $12{\,}877$ genes from fused ranking.

The top-ranked GF-IDF genes were enriched for terms on extracellular matrix (ECM) and structure organisation, as well as collagen fibril organisation. Developmental processes such as skin, eye, and circulatory system were also prominent. Terms related to adhesion and epithelial to mesenchymal transition were additionally enriched. Disease enrichment highlighted strong associations with Ehlers-Danlos Syndrome and related phenotypes. These included connective tissue abnormalities, scoliosis, flat foot, and skin-related traits such as soft or velvety skin. This indicates that the literature-derived gene ranking reflects structural and phenotypic aspects of hEDS/HSD.

The DEGs set showed enrichment for processes related to cell state and activity. These included cellular responses and regulation of cell migration, cell proliferation, DNA damage response, and apoptotic processes, aligning with the results of the original study [38]. Disease terms were dominated by neoplastic and fibrotic conditions. This pattern suggests that DEGs capture disease-related processes but also general fibroblast activation states.

The fused ranking combined features of both inputs. ECM-related processes remained central. In addition, terms such as osteoblast differentiation, epithelial to mesenchymal transition. Additionally, apoptotic and stress-related terms were enriched. Disease associations included Ehlers-Danlos Syndrome as well as osteogenesis imperfecta, and phenotypes associated with the spine, joints, skin, and fibroid conditions. Compared with the individual inputs, the fused set showed a connected tissue disease profile across multiple biological levels, ranging from cellular to phenotypical features.

In this use case, we combined disease-specific knowledge extracted directly from unstructured text with experimental gene activity. We used rank-based aggregation without relying on predefined gene sets or known disease genes. We showed that by fusing literature-derived with expression data, we obtain a gene set that captures a disease profile spanning multiple levels of the disease.

### Use case 3: functional interpretation

**Figure 11:**
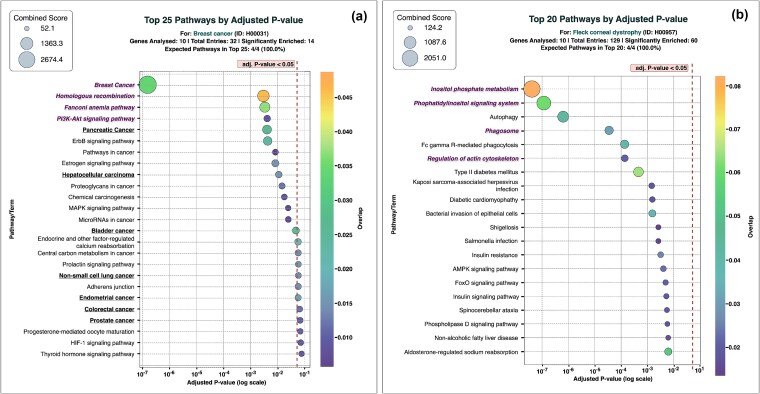
Use case 3: ORA. Bubble plots of the top enriched pathways for two representative diseases. Bubble size represents the combined score, colour indicates pathway overlap, and the x-axis shows adjusted $P$-values on a log scale. The dashed vertical line marks the significance threshold at adjusted $P=.05$. (a) Breast cancer: all four KEGG-expected pathways (Breast cancer, Homologous recombination, Fanconi anaemia pathway, and PI3K-Akt signalling pathway) were recovered as the four most significantly enriched pathways, with Breast cancer showing the strongest enrichment. Additional enriched pathways include ErbB signalling and oestrogen signalling, alongside multiple cancer-related network modules (e.g. bladder cancer, non-small cell lung cancer). (b) Fleck corneal dystrophy: all four KEGG-expected pathways (Inositol phosphate metabolism, Phosphatidylinositol signalling system, Phagosome, and Regulation of actin cytoskeleton) were recovered among the most significantly enriched pathways. Additional enriched terms include autophagy, phagosome-related processes, and metabolic signalling pathways such as AMPK and FoxO signalling.

In Section Performance evaluation, we use gene rankings derived from the literature with a subsequent ORA for performance evaluation. Here, we want to expand this by showcasing the most enriched KEGG pathways for (i) a highly studied (breast cancer) and (ii) a rare disease (Fleck Corneal Dystrophy) (Fig. 11). Based on the top 10 genes according to GF-IDF that were retrieved with pathXcite for breast cancer, we could identify $32$ KEGG entries among which 14 were significantly enriched. The highest ranked KEGG pathway was *Breast Cancer*, highlighting the approach’s precision. The KEGG database also includes network modules that are manually curated higher-order links connecting shared molecular mechanisms and cancers, thus placing the term in a broader global context. Here, of these $22$ network terms, we found eight in our enrichment that reflect common oncogenic processes shared between these cancers.

Beyond the KEGG-listed pathways, the analysis also retrieved the ErbB signalling pathway and the oestrogen signalling pathway. These are well known for their roles in HER2-driven and hormone receptor-positive breast cancer. This demonstrates that such literature-derived genes can also result in the enrichment of relevant but non-curated pathways (within KEGG) [41, 42].

In contrast to breast cancer, we had only $14$ retrieved articles for the rare and under-studied disorder *Fleck Corneal Dystrophy*, making it a challenging use case. Despite limited data, pathXcite could successfully retrieve all four KEGG-listed pathways, including Inositol phosphate metabolism, Phosphatidylinositol signalling system, Phagosome, and Regulation of actin cytoskeleton (Fig. 11b). Notably, additional pathways, such as AMPK signalling and FOXO signalling, were enriched, pointing to potential metabolic and oxidative stress-related mechanisms [43, 44]. PathXcite could hence also support researchers in hypothesis generation in settings where even scarce literature provides additional mechanistic context beyond curated annotations.

### Use case 4: network-based analyses

Across gene set libraries such as KEGG, or DisGeNET, there is no universal standard for what constitutes a “term” [25, 40, 45]. While some resources emphasise a core set of driver genes embedded in well-characterised molecular mechanisms, others adopt a broader set of associated genes. Consequently, diseases like breast cancer can be represented in various ways, either as multiple, finely defined subterms reflecting specific stages, or as more general signatures. KEGG, a comparatively small collection of terms (320 terms in KEGG Human 2021), is built around known molecular interactions and regulatory relationships. As an example, it includes a single breast cancer-related pathway comprising $147$ genes [25, 45]. In contrast, DisGeNet links diseases to genes based on multi-modal and expert-reviewed sources. In the context of breast cancer, this results in over $50$ term definitions and in total $>5580$ genes associated with this cancer and its subdefinitions [40].

The most suitable definition is inherently tied to the intended application. However, when investigating highly specialised contexts, existing curated gene sets may either lack sufficient specificity or fail to adequately capture the biological processes of interest. To address this limitation, this use case integrates prior, curated knowledge with literature-derived evidence in a network-based framework. Specifically, we projected gene sets onto protein–protein interaction networks combining curated pathway genes with gene lists generated through targeted literature mining using pathXcite. This approach enables a rapid assessment of whether existing knowledge sufficiently captures the biological context or whether additional mechanisms emerge from the literature.

Concretely, we selected all genes from the KEGG term “breast cancer” and complemented them with an equally sized set of top-ranked genes based on GF-IDF scores derived from a pathXcite query “‘breast cancer’[title]” (PMC).

For each pair of genes, we evaluated the presence of interactions using the OmniPath database, adding an edge whenever a documented interaction was identified [46].

First, we performed the network construction procedure on the original literature-derived and KEGG-derived gene sets separately. Here, we obtained a literature-derived network graph of $140$ nodes connected by $1{}098$ edges ($7$ unconnected nodes), and a KEGG-derived network graph of $147$ nodes connected by $1{}572$ edges. Comparing both graphs, we found $19$ nodes and $72$ edges which appeared in both original topologies.

Then, we fused the original gene sets to produce a fused network graph to inspect whether the integration of both original gene sets could uncover nodes or edges which were not included in the separate networks. This produced a fused network of $269$ gene nodes connected by $3{}403$ edges. Here, six nodes were unconnected. Through fusion of the original gene sets, one additional node was now connected (*ALKBH5*). Also, $999$ additional edges were uncovered by fusion. The literature-derived set contributed $121$ unique nodes and $928$ edges, while KEGG contributed $128$ nodes and $1{}405$ edges.

Importantly, the literature-derived gene set captured biologically relevant genes not present in the KEGG pathway. For example, PARP1 is included, a key mediator of synthetic lethality in combination with BRCA1 in breast cancer [47]. Hence, integrating literature-derived gene sets can extend beyond curated pathway knowledge and provide additional insights into disease-relevant mechanisms. Also, this experiment exemplifies how fusion of two gene lists from different modalities can produce additional connections when integrated into a protein–protein network graph.

For comparison, the same approach was performed with literature-derived genes ($962$ genes from $250$ articles) and genes associated with breast cancer within the DisGeNET dataset ($5{}583$ unified from $52$ single terms). Through the creation of a fused network, $16$ newly connected nodes and $2{}486$ additional edges were found. While both original sets differed strongly in size, $203$ nodes and $1568$ edges were contributed by the integration of literature-derived genes.

In both of the network analyses (with KEGG and DisGeNet), the set of additionally uncovered genes through network fusion contained the gene ALKBH7, the latter also identified ALKBH1. This further demonstrates the added value through fusion, as the ALKBH family members were previously identified as biomarkers and prognostic factors for breast cancer [48]. These were not present in the curated set-derived networks but emerged after fusion with literature-derived genes.

Other examples of genes which emerged from literature include the gene CENPI, which was identified as one of $49$ hub genes of breast cancer in a study from 2019, yet the only association within DisGeNET is to autoimmune diseases so far [49]. In summary, integrating literature-derived gene lists into protein–protein interaction networks can complement curated resources such as KEGG and DisGeNET. Curated databases offer structured and reliable knowledge. However, they are limited by predefined scopes and update cycles.

Literature-derived gene sets capture more dynamic and context-specific information. They include emerging findings and less well-characterised, but potentially relevant mechanisms. When mapped onto interaction networks, these genes add new nodes and edges, which expands network coverage and increases connectivity.

Fusion enables interactions between genes originating from different sources, thereby revealing cross-context connectivity not observable within isolated or curated gene sets. It helps identify new functional modules and potential mechanisms, which even large databases might not yet fully capture.

### Use case 5: disease clustering

This use case demonstrates how diseases can be clustered based on shared literature-informed enrichment profiles rather than traditional classifications, such as organ system (e.g. cardiovascular versus respiratory) or aetiology (e.g. infectious versus genetic).

From the $1307$ diseases analysed in Section Performance evaluation, we selected the $70$ diseases with a dedicated KEGG disease-specific pathway (e.g. Breast Cancer [H00031] and its corresponding pathway hsa05224). This ensured a curated, diverse and still biologically interpretable set of well-known diseases for clustering. Gene list construction followed the approach as described before. As we found that already a moderate number of top-ranking genes by GF-IDF provides a sufficient signal for enrichment, we selected the $70$ highest-scoring genes from the mined literature for each disease. These gene lists were then used for ORA against five different gene set libraries representing pathways, ontologies, or disease associations:

- Pathways: KEGG (2022) [45], Reactome [26], WikiPathways (Human, 2024) [50],
- Ontologies: GO Biological Process (2025) [27],
- Disease associations: DisGeNET [40].

To allow for a fair comparison across diseases, we retained the top $50$ most enriched terms (pathways, processes, or diseases) ranked by adjusted $P$-value in increasing order, which we denote as the enrichment profiles $r_{d}$ for each disease $d$. Terms not present among the top $K=50$ results for a given disease were assigned a rank of $K+1$ to ensure equal-sized ranks across diseases.

Pairwise distances between diseases $d_{i}$ and $d_{j}$ were then computed as $1 - \rho _{r_{d_{i}}, r_{d_{j}}}$, where $\rho _{r_{d_{i}}, r_{d_{j}}}$ is the Spearman rank correlation coefficient between their enrichment profiles. The coefficient was calculated using the standard covariance-based formulation:


(5) \begin{eqnarray*}& \rho_{r_{d_{i}},r_{d_{j}}} = \frac{ \sum_{k=1}^{K} \left( r_{d_{i}}(k) - \bar{r}_{d_{i}} \right)\left( r_{d_{j}}(k) - \bar{r}_{d_{j}} \right) } { \sqrt{ \sum_{k=1}^{K} \left( r_{d_{i}}(k) - \bar{r}_{d_{i}} \right)^{2}} \sqrt{ \sum_{k=1}^{K} \left( r_{d_{j}}(k) - \bar{r}_{d_{j}} \right)^{2}} } \,,\end{eqnarray*}

where $\bar{r}_{d_{i}}$ and $\bar{r}_{d_{j}}$ denote the mean ranks in the respective profiles.

**Figure 12:**
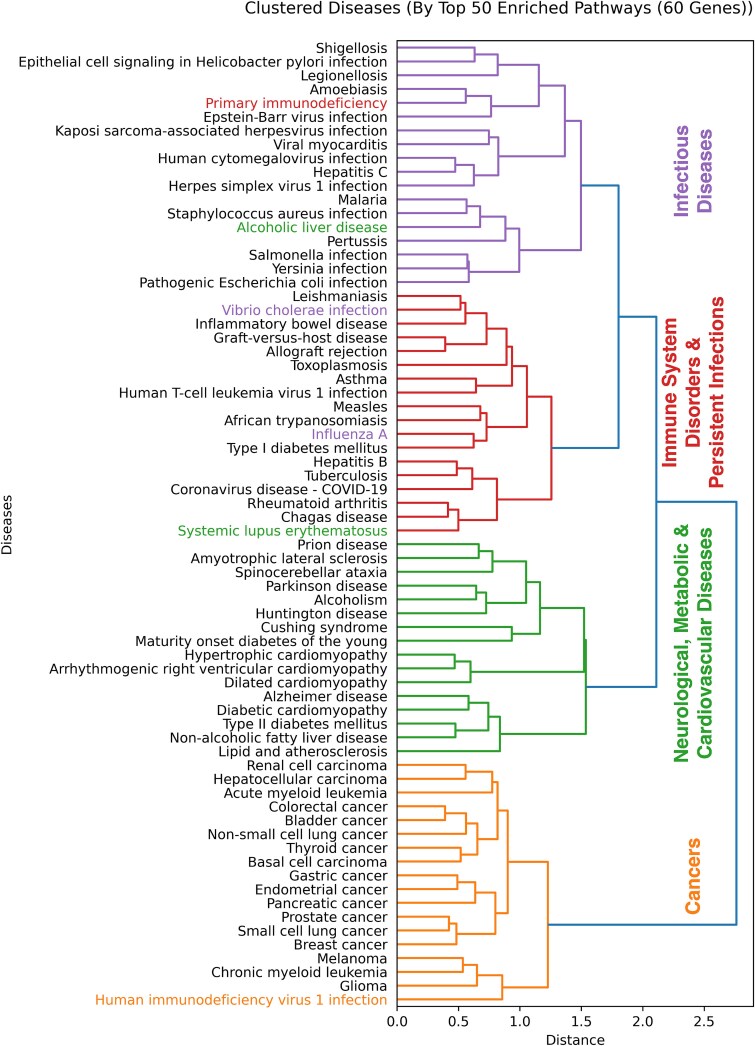
Use case 5: disease clustering. Hierarchical clustering of $70$ diseases based on the top $50$ enriched KEGG pathways. Diseases were grouped using Ward’s method, resulting in four primary clusters: neurological and metabolic diseases, immune and infectious diseases, tropical infections and autoimmune diseases, and oncological diseases. The dendrogram highlights expected disease groupings and reveals potential shared mechanisms across clusters. For representation purposes, the colour threshold was set to $1.6$.

Agglomerative hierarchical clustering was then applied to these distance matrices using the Ward’s minimum variance method as the linkage criterion [51, 52]. This procedure was carried out independently for each library, yielding five disease clusterings based solely on their ORA-derived ranking profiles. Across all gene set libraries, hierarchical clustering produced biologically meaningful disease groupings, broadly aligning with four major categories: (i) Infectious Diseases, (ii) Immune System Disorders & Viral-Persistent Diseases, (iii) Neurological, Metabolic, & Cardiovascular Disorders, and (iv) Cancers (Fig. 12). To quantify the degree to which different libraries produced comparable groupings, we computed the pairwise normalised mutual information (NMI) between all clustering outcomes [53].

**Figure 13:**
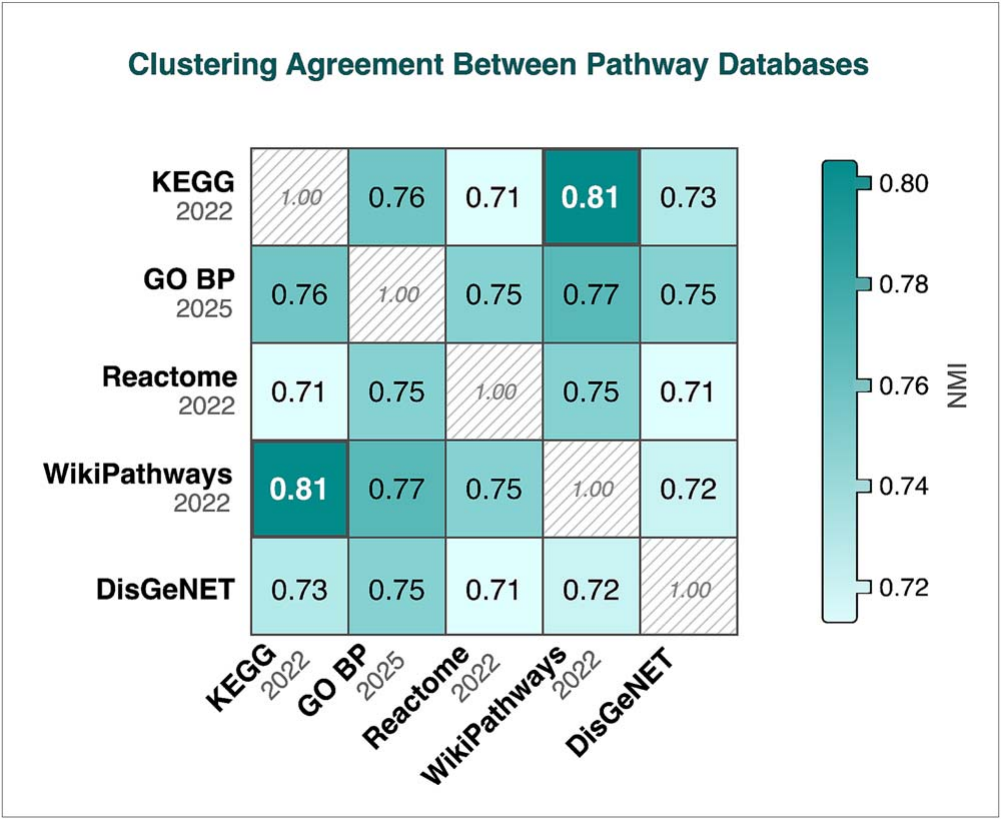
Use case 5: disease clustering. Pairwise NMI between disease clusterings derived from five different gene set libraries and the resulting enrichment profiles. Each value shows the similarity between two clusterings, with $0$ representing no shared structure and $1$ indicating identical groupings. NMI values ranged from $0.71$ to $0.81$, showing that disease clusters based on literature-derived pathway enrichment profiles are largely consistent across gene set libraries. The highest agreement was observed between KEGG and WikiPathways, while Reactome showed slightly more deviation from the others, likely reflecting differences in term granularity and coverage.

In this setting, NMI values range from $0$ (independent clusterings) to $1$ (identical clusterings), providing a scale-independent measure of concordance. For our comparison, we obtained NMI values ranged from $0.71$ to $0.81$ (Fig. 13), suggesting that while each gene set library introduced minor variations, overall clustering structures remained largely stable. The highest agreement was observed between KEGG and WikiPathways (NMI = $0.81$), while Reactome exhibited slightly lower consistency with KEGG (NMI = $0.71$). This indicates that clustering based on literature-derived enrichment profiles is robust across different gene set libraries, with minor variations likely reflecting their differences in granularity and semantic definitions of gene sets/terms. While most diseases clustered as expected, some appeared as outliers. To examine whether unexpected diseases in a cluster show non-random biological similarity, we assessed pathway-level overlap between each outlier and its closest neighbours. We used hypergeometric $P$-values for shared pathways $k$ (overlap $o$) to determine statistical significance ($P <.05$). This allows us to distinguish biologically meaningful relationships from ranking similarity artefacts.

For example, the term *Alcoholic liver disease* was an unexpected member of the cluster *Infectious Diseases* which primarily grouped bacterial, viral, and parasitic infections. Despite being a metabolic disorder, *Alcoholic liver disease* showed strong pathway similarity to both *Malaria*(*o*=$28$/$50$, $P<.0001$) and *Staphylococcus aureus infection* (*o*=$30$/$50$, $P<.00001$). Shared enrichment included the *HIF-1 signalling pathway*, activated during liver hypoxia in alcoholic liver disease and in severe malaria due to red blood cell destruction. Furthermore, the shared pathways *African trypanosomiasis, Tuberculosis*, and *AGE-RAGE signalling pathway* reflect common immune activation, oxidative stress, and vascular dysfunction [54–57]. When checking the other outliers, similar biological and evidence-based justifications can be identified, thus supporting disease relationships rather than misclassifications. One notable exception was *Primary immunodeficiency*, which clustered with infections due to immune dysregulation but lacked significant pathway overlap. Overall, these findings show that enrichment profile clustering can reveal non-traditional disease relationships and that clustering outcomes remain largely consistent across different curated gene set libraries.

## Discussion

The biomedical literature encompasses a vast and largely unstructured body of knowledge describing molecular mechanisms in diverse biological contexts. Gene mentions within these texts can be aggregated into topic-specific gene lists that reflect the molecular entities most strongly associated with a topic [12, 14, 15]. To retrieve gene lists from the literature, machine learning-based systems have been shown to outperform dictionary- and rule-based approaches, a finding we confirmed using the BioRED corpus [34], which comprises 1180 manually annotated and normalised gene mentions from 100 PubMed articles. As literature-derived gene collections are intended to capture the full breadth of topic-associated molecular signals, this improved coverage motivated our adoption of a machine learning-based retrieval approach via PubTator$^{3}$ [24] within pathXcite.

Previous studies have analysed the literature for functional analysis, but typically rely on custom, task-specific pipelines [58–61]. These approaches often address isolated steps and do not integrate all steps of corpus definition, gene extraction, prioritisation, and downstream analysis within a reproducible workflow (Fig. 3). PathXcite addresses this by providing an integrated and transparent system for constructing and analysing literature-derived gene lists.

After retrieving these gene lists from the literature, a critical step is the prioritisation of these genes for further analysis. Many existing approaches rely on frequency-based methods [19]. However, annotation frequency alone can bias results towards genes that are extensively studied and frequently cited, regardless of their specific relevance to the topic of interest.

Our approach accounts for the aforementioned biases by balancing gene frequency with contextual specificity. Although we recommend the application of this adapted ranking strategy, referred to as GF-IDF, pathXcite also retains the option for users to apply a more conservative frequency-based ranking.

In various benchmarking experiments, we challenged our GF-IDF ranking method of genes derived from the literature and showed that it outperforms the raw frequency-based selection (Fig. 6).

Furthermore, in scalability experiments (Fig. 7) we showed that literature-derived gene lists are corpus-dependent: small corpora may lack coverage, whereas very large corpora introduce noise and bias towards generic gene signal, especially when list sizes are fixed. Here, the GF-IDF metric showed to accommodate larger gene sets without introducing significant noise. These findings underscore the importance of explicit ranking strategies and transparent corpus definition when transforming textual evidence into analyseable gene sets. In a series of use-case examples, we further demonstrate how literature-derived gene lists, such as those generated by pathXcite, can be leveraged to uncover biological mechanisms and relationships by integrating distributed, unstructured knowledge into a global multi-omics context. In this way, pathXcite provides a framework for structuring and analysing gene-centric information extracted from biomedical literature.

## Conclusion

We presented pathXcite, an integrated framework for constructing and prioritising literature-derived gene lists in a transparent and reproducible manner. By combining corpus definition, automated gene extraction, and context-sensitive ranking, the platform enables the systematic transformation of biomedical text into structured gene lists suitable for downstream analyses.

**Key Points**

- PathXcite is an integrated graphical interface that transforms biomedical literature on a topic into interpretable gene lists.
- We introduce GF-IDF, a new context-aware ranking method for gene lists, called Gene Frequency-Inverse Document Frequency (GF-IDF).
- The application of the GF-IDF ranking metric outperformed other gene selection methods and is less sensitive to gene counts.
- We provide diverse use case examples from feature selection, enrichment analysis, network approaches, and rank fusion to show how literature-derived gene lists can uncover new biological insights.

## Supplementary Material

supplement_bbag348

## Data Availability

All data applied are publicly available and referenced accordingly. The code to reproduce the analyses is available at https://github.com/sysbio-bioinf/pathXcite">https://github.com/sysbio-bioinf/pathXcite and https://doi.org/10.5281/zenodo.17417215.

## Credit statement

Marietta Hamberger: Conceptualisation, Methodology, Software, Formal analysis, Writing—original draft. Silke D. Werle: Formal analysis, Writing—original draft. Johann M. Kraus: Supervision, Formal analysis, Writing—review & editing. Hans A. Kestler: Conceptualisation, Supervision, Project administration, Funding acquisition, Writing—review & editing.

## References

[1] Hagenauer MH, Sannah Y, Hebda-Bauer EK et al. Resource: A curated database of brain-related functional gene sets (Brain.GMT). MethodsX 2024;13:102788. 10.1016/j.mex.2024.102788 PMC11267058 39049932

[2] Hemedan AA, Niarakis A, Schneider R et al. Boolean modelling as a logic-based dynamic approach in systems medicine. *Comput Struct Biotechnol J* 2022;20:3161–72. 10.1016/j.csbj.2022.06.035 35782730 PMC9234349

[3] Sundermann B, Olde lütke Beverborg M, Pfleiderer B. Toward literature-based feature selection for diagnostic classification: a meta-analysis of resting-state fMRI in depression. *Front Hum Neurosci* 2014;8:2014. 10.3389/fnhum.2014.00692 PMC4159995 25309382

[4] Tan J, Huyck M, Hu D et al. ADAGE signature analysis: differential expression analysis with data-defined gene sets. *BMC Bioinformatics* 2017;18:512. 10.1186/s12859-017-1905-4 29166858 PMC5700673

[5] Ahmad PN, Shah AM, Lee K. A review on electronic health record text-mining for biomedical name entity recognition in healthcare domain. *Healthcare* 2023;11:1268. 10.3390/healthcare11091268 37174810 PMC10178605

[6] Leser U, Hakenberg J. What makes a gene name? Named entity recognition in the biomedical literature. *Brief Bioinform* 2005;6:357–69. 10.1093/bib/6.4.357 16420734

[7] Perera N, Dehmer M, Emmert-Streib F. Named entity recognition and relation detection for biomedical information extraction. *Front Cell Dev Biol* 2020;8:673. 10.3389/fcell.2020.00673 32984300 PMC7485218

[8] Clarke DJB, Marino GB, Deng EZ et al. Rummagene: massive mining of gene sets from supporting materials of biomedical research publications. *Commun Biol* 2024;7:482. 10.1038/s42003-024-06177-7 38643247 PMC11032387

[9] Ofer D, Brandes N, Linial M. The language of proteins: NLP, machine learning & protein sequences. *Comput Struct Biotechnol J* 2021;19:1750–8. 10.1016/j.csbj.2021.03.022 33897979 PMC8050421

[10] Shetty P, Udhayakumar R, Patil A et al. Application of natural language processing (NLP) in machine learning. In:*3rd International Conference on Advancement in Electronics & Communication Engineering (AECE)*, pp. 949–57. Ghaziabad, India: IEEE, 2023.

[11] Wu S, Roberts K, Datta S et al. Deep learning in clinical natural language processing: a methodical review. *J Am Med Inform Assoc* 2020;27:457–70. 10.1093/jamia/ocz200 31794016 PMC7025365

[12] Baltoumas FA, Zafeiropoulou S, Karatzas E et al. OnTheFly2.0: a text-mining web application for automated biomedical entity recognition, document annotation, network and functional enrichment analysis. *NAR Genom Bioinform* 2021;3:lqab090. 10.1093/nargab/lqab090 34632381 PMC8494211

[13] Fontaine J-F, Priller F, Barbosa-Silva A et al. Génie: literature-based gene prioritization at multi genomic scale. *Nucleic Acids Res* 2011;39:W455–61. 10.1093/nar/gkr246 21609954 PMC3125729

[14] Karatzas E, Baltoumas FA, Aplakidou E et al. Flame (v2.0): advanced integration and interpretation of functional enrichment results from multiple sources. Bioinformatics 2023;39:btad490. 10.1093/bioinformatics/btad490 PMC10423032 37540207

[15] Wang J-H, Zhao L-F, Weng H-F et al. GenCLiP 3: mining human genes’ functions and regulatory networks from PubMed based on co-occurrences and natural language processing. *Bioinformatics* 2019;36:1973–5. 10.1093/bioinformatics/btz807 31681951

[16] Lachmann A, Schilder BM, Wojciechowicz ML et al. Geneshot: search engine for ranking genes from arbitrary text queries. *Nucleic Acids Res* 2019;47:W571–7. 10.1093/nar/gkz393 31114885 PMC6602493

[17] Özgür A, Vu T, Erkan G et al. Identifying gene-disease associations using centrality on a literature mined gene-interaction network. *Bioinformatics* 2008;24:i277–85. 10.1093/bioinformatics/btn182 18586725 PMC2718658

[18] Natarajan J, Ganapathy J. Functional gene clustering via gene annotation sentences, MeSH and GO keywords from biomedical literature. *Bioinformation* 2007;2:185–93. 10.6026/97320630002185 18305827 PMC2241933

[19] Baeza-Yates R, Ribeiro-Neto B. Modern Information Retrieval, Vol. 463. New York, NY, USA: ACM Press, 1999.

[20] Salton G, Wong A, Yang C-S. A vector space model for automatic indexing. *Commun ACM* 1975;18:613–20. 10.1145/361219.361220

[21] Sparck Jones K . A statistical interpretation of term specificity and its application in retrieval. *J Doc* 1972;28:11–21. 10.1108/eb026526

[22] Sayers E . A general introduction to the E-utilities. In: *Entrez Programming Utilities Help [Internet]*. Bethesda (MD): National Center for Biotechnology Information (US), 2010.

[23] Wei C-H, Allot A, Leaman R et al. PubTator central: automated concept annotation for biomedical full text articles. *Nucleic Acids Res* 2019;47:W587–93. 10.1093/nar/gkz389 31114887 PMC6602571

[24] Wei C-H, Allot A, Lai P-T et al. PubTator 3.0: an AI-powered literature resource for unlocking biomedical knowledge. *Nucleic Acids Res* 2024;52:W540–6. 10.1093/nar/gkae235 38572754 PMC11223843

[25] Kanehisa M, Furumichi M, Sato Y et al. KEGG: biological systems database as a model of the real world. *Nucleic Acids Res* 2025;53:D672–7. 10.1093/nar/gkae909 39417505 PMC11701520

[26] Milacic M, Beavers D, Conley P et al. The Reactome pathway knowledgebase 2024. *Nucleic Acids Res* 2024;52:D672–8. 10.1093/nar/gkad1025 37941124 PMC10767911

[27] The Gene Ontology Consortium . The gene ontology knowledgebase in 2023. *Genetics* 2023;224:iyad031. 36866529 10.1093/genetics/iyad031 PMC10158837

[28] Gargano MA, Matentzoglu N, Coleman B et al. The human phenotype ontology in 2024: phenotypes around the world. *Nucleic Acids Res* 2023;52:D1333–46. 10.1093/nar/gkad1005 PMC10767975 37953324

[29] Yoo M, Shin J, Kim J et al. DSigDB: drug signatures database for gene set analysis. *Bioinformatics* 2015;31:3069–71. 10.1093/bioinformatics/btv313 25990557 PMC4668778

[30] Luo L, Wei C-H, Lai P-T et al. AIONER: all-in-one scheme-based biomedical named entity recognition using deep learning. *Bioinformatics* 2023;39:btad310. 10.1093/bioinformatics/btad310 37171899 PMC10212279

[31] Wei C-H, Luo L, Islamaj R et al. GNorm2: an improved gene name recognition and normalization system. *Bioinformatics* 2023;39:btad599. 10.1093/bioinformatics/btad599 37878810 PMC10612401

[32] Pafilis E, Buttigieg PL, Ferrell B et al. EXTRACT: interactive extraction of environment metadata and term suggestion for metagenomic sample annotation. *Database* 2016;2016:baw005. 26896844 10.1093/database/baw005 PMC4761108

[33] Seow WL, Chaturvedi I, Hogarth A et al. A review of named entity recognition: from learning methods to modelling paradigms and tasks. Artif Intell Rev 2025;58:315. 10.1007/s10462-025-11321-8

[34] Luo L, Lai P-T, Wei C-H et al. BioRED: a rich biomedical relation extraction dataset. *Brief Bioinform* 2022;23:bbac282. 10.1093/bib/bbac282 35849818 PMC9487702

[35] Dasarathy BV . Nearest Neighbor (NN) Norms: NN Pattern Classification Techniques. Los Alamitos, CA: IEEE Computer Society Press, 1991.

[36] Burkovski A, Lausser L, Kraus JM et al. Rank aggregation for candidate gene identification. In: Spiliopoulou M, Schmidt-Thieme L, Janning R (eds.), Data Analysis, Machine Learning and Knowledge Discovery, pp. 285–93. Cham, Switzerland: Springer International Publishing; 2013.

[37] Scicluna K, Formosa MM, Farrugia R et al. Hypermobile Ehlers–Danlos syndrome: a review and a critical appraisal of published genetic research to date. *Clin Genet* 2022;101:20–31. 10.1111/cge.14026 34219226

[38] Ritelli M, Chiarelli N, Cinquina V et al. RNA-Seq of dermal fibroblasts from patients with hypermobile Ehlers–Danlos syndrome and hypermobility Spectrum disorders supports their categorization as a single entity with involvement of extracellular matrix degrading and proinflammatory Pathomechanisms. *Cells* 2022;11:4040. 10.3390/cells11244040 36552803 PMC9777098

[39] Borda JC d . Mémoire Sur les élections au scrutin. *Histoire de l’Académie Royale des Sciences*, Jg 1784;1781:657–65.

[40] Piñero J, Ramírez-Anguita JM, Saüch-Pitarch J et al. The DisGeNET knowledge platform for disease genomics: 2019 update. *Nucleic Acids Res* 2020;48:D845–55. 10.1093/nar/gkz1021 31680165 PMC7145631

[41] Drago JZ, Ferraro E, Abuhadra N et al. Beyond HER2: targeting the ErbB receptor family in breast cancer. *Cancer Treat Rev* 2022;109:102436. 10.1016/j.ctrv.2022.102436 35870237 PMC10478787

[42] Pegram M, Jackisch C, Johnston SRD. Estrogen/HER2 receptor crosstalk in breast cancer: combination therapies to improve outcomes for patients with hormone receptor-positive/HER2-positive breast cancer. *NPJ Breast Cancer* 2023;9:45. 10.1038/s41523-023-00533-2 37258523 PMC10232442

[43] Chen J, Ou Q, Wang Z et al. Small-molecule induction promotes corneal endothelial cell differentiation from human iPS cells. *Front Bioeng Biotechnol* 2021;9:788987. 10.3389/fbioe.2021.788987 34976977 PMC8714889

[44] Du S, Zheng H. Role of FoxO transcription factors in aging and age-related metabolic and neurodegenerative diseases. *Cell Biosci* 2021;11:188. 10.1186/s13578-021-00700-7 34727995 PMC8561869

[45] Kanehisa M, Furumichi M, Sato Y et al. KEGG for taxonomy-based analysis of pathways and genomes. *Nucleic Acids Res* 2023;51:D587–92. 10.1093/nar/gkac963 36300620 PMC9825424

[46] Türei D, Korcsmáros T, Saez-Rodriguez J. OmniPath: guidelines and gateway for literature-curated signaling pathway resources. *Nat Methods* 2016;13:966–7. 10.1038/nmeth.4077 27898060

[47] Cong K, Peng M, Kousholt AN et al. Replication gaps are a key determinant of PARP inhibitor synthetic lethality with BRCA deficiency. *Mol Cell* 2021;81:3128–3144.e7. 10.1016/j.molcel.2021.06.011 34216544 PMC9089372

[48] Chen H, Zhou L, Li J et al. ALKBH family members as novel biomarkers and prognostic factors in human breast cancer. *Aging* 2022;14:6579–93. 10.18632/aging.204231 35980268 PMC9467415

[49] Fu Y, Zhou Q-Z, Zhang X-L et al. Identification of hub genes using co-expression network analysis in breast cancer as a tool to predict different stages. *Med Sci Monit* 2019;25:8873–90. 10.12659/MSM.919046 31758680 PMC6886326

[50] Agrawal A, Balci H, Hanspers K et al. Wikipathways 2024: next generation pathway database. *Nucleic Acids Res* 2024;52:D679–89. 10.1093/nar/gkad960 37941138 PMC10767877

[51] Ward JHJr . Hierarchical grouping to optimize an objective function. *J Am Stat Assoc* 1963;58:236–44. 10.1080/01621459.1963.10500845

[52] Webber W, Moffat A, Zobel J. A similarity measure for indefinite rankings. *ACM Trans Inf Syst* 2010;28:1–38. 10.1145/1852102.1852106

[53] Strehl A, Ghosh J. Cluster ensembles – a knowledge reuse framework for combining multiple partitions. *J Mach Learn Res* 2002;3:583–617.

[54] Ballard HS . The hematological complications of alcoholism. *Alcohol Health Res World* 1997;21:42–52. 15706762 PMC6826798

[55] Krücken J, Mehnert LI, Dkhil MA et al. Massive destruction of malaria-parasitized red blood cells despite spleen closure. *Infect Immun* 2005;73:6390–8. 10.1128/IAI.73.10.6390-6398.2005 16177310 PMC1230986

[56] Nath B, Levin I, Csak T et al. Hepatocyte-specific hypoxia-inducible factor-1$\alpha$ is a determinant of lipid accumulation and liver injury in alcohol-induced steatosis in mice. *Hepatology* 2011;53:1526–37. 10.1002/hep.24256 21520168 PMC3104403

[57] Wei H, Xie A, Li J et al. PD-1+ CD4 T cell immune response is mediated by HIF-1α/NFATc1 pathway after P. Yoelii infection. Front Immunol 2022;13:942862. 10.3389/fimmu.2022.942862 PMC9449323 36091043

[58] Du Y, He L, Ye X et al. To explore the molecular mechanism of acupuncture alleviating inflammation and treating obesity based on text mining. *Biomed Res Int* 2022;2022:3133096. 10.1155/2022/3133096 36105933 PMC9467717

[59] Hu D, Lin Z, Jiang J et al. Identification of key genes and molecular pathways in keratoconus: integrating text mining and bioinformatics analysis. *Biomed Res Int* 2022;2022:4740141. 10.1155/2022/4740141 36051483 PMC9427295

[60] Rong T, Rong S, Zeng Y et al. Analysis of gene function enrichment and drug treatment of oral mucositis after radiotherapy and chemotherapy for head and neck tumor based on computer text mining. *Adv Clin Med* 2020;10:1367–75. 10.12677/ACM.2020.107206

[61] Wang Y, Zhao B, Yuan X-J. Drug discovery and potential gene and pathway associated with polycystic ovary syndrome through text mining and biomedical databases. *Reprod Dev Med* 2022;7:44–9. 10.1097/RD9.0000000000000035

