# Supplementary material for: Literature-informed gene extraction and ranking for multimodal data fusion
Source: Brief Bioinform. 2026 Jul 5;27(4):bbag348. doi: 10.1093/bib/bbag348 (PMC13333087; doi:10.1093/bib/bbag348)
Supplement: supplement_bbag348 [file supplement_bbag348.pdf]

S1 Supplementary Material

S1.1 Computational Details and Scalability

All use cases were performed on a MacBook Pro (Apple M1 Pro, 10-core CPU, 16-core GPU, 32 GB RAM) running macOS Sequoia 15.

The retrieval of metadata and annotations for a batch of 200 articles required approximately 13 seconds (approximately 0.065 s/article). Most of this time is attributed to API latency and adherence to the rate limits of NCBI E-utilities and PubTator[3] [1-3]. GF-IDF computation and the population of the gene table occurred in less than 100 milliseconds per dataset. As the computation is performed locally, enrichment analyses for more than 1,000 genes took less than 5 seconds to compute and visualise.

References

[1] Baeza-Yates, R. and Ribeiro-Neto, B. (1999). Modern information retrieval, volume 463. ACM press, New York, NY, USA.
[2] Wei, C.-H., Allot, A., Leaman, R., et al. (2019). PubTator central: automated concept annotation for biomedical full text articles. Nucleic Acids Research, 47(W1), W587–W593.
[3] Wei, C.-H., Allot, A., Lai, P.-T., et al. (2024). PubTator 3.0: an AI-powered literature resource for unlocking biomedical knowledge. Nucleic Acids Research, 52(W1), W540–W546.
